# Hospital variation in the risk of infection after hip fracture surgery: a population-based cohort study including 29,598 patients from 2012–2017

**DOI:** 10.1080/17453674.2020.1863688

**Published:** 2020-12-18

**Authors:** Jeppe Damgren Vesterager, Pia Kjaer Kristensen, Irene Petersen, Alma Becic Pedersen

**Affiliations:** a Department of Clinical Epidemiology, Aarhus University Hospital, Aarhus N, Denmark;; b Department of Orthopedic Surgery, Regional Hospital Horsens, Horsens, Denmark;; c epartment of Primary Care and Population health, University College London, London, UK

## Abstract

Background and purpose — Understanding the key drivers of hospital variation in postoperative infections after hip fracture surgery is important for directing quality improvements. Therefore, we investigated variation in the risk of any infection, and subgroups of infections including pneumonia and sepsis after hip fracture surgery.

Methods — In this nationwide population-based cohort study, all Danish patients aged ≥ 65 undergoing surgery for an incident hip fracture from 2012 to 2017 were included. Risk of postoperative infections, based on data from hospital registration (hospital-treated infections) and antibiotic dispensing (community-treated infections), were calculated using multilevel Poisson regression analysis. Hospital variation was evaluated by intra-class coefficient (ICC) and median risk ratio (MRR).

Results — The risk of hospital-treated infection was 15%. The risk of community-treated infection was 24%. The adjusted risk varied between hospitals from 7.8–25% for hospital-treated infection and 16–34% for community-treated infection. The ICC indicated that 19% of the adjusted variance was due to hospital level for hospital-treated infection. The ICC for community-treated infections was 13%. The MRR showed a 2-fold increased risk for the average patient acquiring a hospital-treated infection at the highest risk hospital compared with the lowest risk hospital. For community-treated infection, the MRR was 1.4.

Interpretation — Our results suggest that 20% of infections could be reduced by applying the top performing hospitals’ approach. Nearly a 5th of the variation was at the hospital level. This suggests a more standardized approach to avoid postoperative infection after hip fracture surgery.

Hip fracture is a leading cause of hospital admission among the elderly. The 30-day mortality following hip fracture surgery has been approximately 10% during the last few years in Denmark (Pedersen et al. 2017). Higher mortality after hip fracture has been associated with a range of hospital factors (Kristensen et al. 2016, Sheehan et al. 2016) and patient factors in observational studies (Roche et al. 2005). Furthermore, variation in 30-day mortality after hip fracture surgery has been observed between Danish hospitals, but not fully explained (Kristensen et al. 2019).

Postoperative infection among hip fracture patients is associated with a 3-fold increase in mortality, within 30 days of operation, compared with non-infected patients (Kjørholt et al. 2019a). Additionally, postoperative infections adversely affect quality of life and hospital costs (Shander et al. 2011). The increased risk of infections after hip fracture surgery is a consequence of multiple patient-, surgery-, and hospital-related factors (Taylor and Oppenheim 1998, Poh and Lingaraj 2013). In the past decade, the 30-day cumulative incidence of postoperative infection after hip fracture has increased substantially in Denmark, reaching 14% in 2015–2016 (Kjørholt et al. 2019b), suggesting room for quality improvement.

Postoperative infections could be a relevant quality performance measure for ranking hospitals as good treatment, rehabilitation, and care of hip fracture patients should reduce postoperative infections. No previous studies have investigated the hospital variation in postoperative infections among hip fracture patients. However, in order to interpret hospital variation in postoperative infections it is important to understand the relative contributions of patient and healthcare factors. Multilevel models can estimate and separate the relative contribution of the hospital context (hospital level) and patient characteristics (patient level) to the total between-hospital variation in the infections. Thus, studying hospital variation in postoperative infections using multilevel models is an important step towards understanding the key drivers of high infection risk in general and implementation of targeted prevention strategies

We investigated the variation between hospitals in the risk of infection within 30 days of hip fracture surgery.

## Methods

### Study design

The study was designed as a cohort study, with surgery-performing hospitals being the exposure and postoperative infection being the outcome. The study was based on data from prospectively collected nationwide population-based medical registries in Denmark.

### Setting and participants

The medical registries used encompasses the entire Danish population. The Danish healthcare system is tax-supported with free access to care (Schmidt et al. 2019). All patients admitted to hospital with a hip fracture from January 1, 2013 until December 1, 2017 were included (n = 29,937). Patients with incorrect recording of time, meaning patients registered to be operated on before admission, were excluded (n = 127). To avoid imprecise estimates, hospitals that performed less than 15 hip fracture surgeries per year (7 hospitals and 74 patients) or no longer performed hip fracture surgery (1 hospital and 138 patients) were excluded. The final study cohort included 29,598 patients treated at 23 hospitals (Figure 1, see Supplementary data). Exposure was hospitals performing surgery, outcomes were any hospital-treated infection, sepsis, pneumonia, and community-treated hospital within 30 days of operation.

### Variables

The primary outcome was hospital-treated infections, identified from the Danish National Patient Registry (DNPR) based on ICD-10 codes (Table 1, see Supplementary data). The list of infections included chronic and more rare infections, so as also to detect possible flare-up in already ongoing infections. We excluded urinary tract infections (UTI) because of the high risk of different registration praxis among hospitals. First, there is no economic benefit in the coding of UTI for the department. Second, elderly patients with UTI often have persistent symptomless bacteriuria, which may cause a positive urinary culture without any symptoms (Gavazzi and Krause 2002). The secondary outcomes were 2 of the most common subtypes of hospital-treated infection: pneumonia and sepsis. Table 2 (see Supplementary data) shows the frequency of all infections.

Community-treated infections were identified from the Danish National Health Service Prescription Database (DNHSPD) based on ATC codes (Table 3, see Supplementary data) and defined as at least 1 dispensing of any antibiotic within 30 days of surgery.

### Covariates

To account for hospital case mix, we collected several well-established prognostic factors known to increase infection risk (Poh and Lingaraj 2013).

Comorbidity was summarized according to the Charlson Comorbidity Index (CCI). From the DNPR, ICD-10 codes were used to identify CCI (Table 4, see Supplementary data). Data on age, sex, BMI, surgery delay, and surgery type was obtained from the Danish Multidisciplinary Hip Fracture Registry (DMHFR).

See Figure 2 (in Supplementary data) for a chart of the study design.

### Data sources

The Danish Civil Registration System (DCRS) has assigned to all residents in Denmark a unique 10-digit personal identification number at birth or upon immigration since 1968. This number encodes age, sex, and date of birth. It is recorded at all contacts with the healthcare system. Therefore, an unambiguous record linkage between all medical registers in the population is possible (Schmidt et al. 2019).

The DMHFR is a nationwide clinical quality registry on all patients aged ≥ 65 years operated on at Danish hospitals with a medial (S720), pertrochanteric (S721), or subtrochanteric (S722) femoral fracture since 2003 (Kristensen et al. 2020, Hjelholt et al. 2020).

The DNPR has registered all non-psychiatric inpatient hospital admissions since 1977 and all hospital outpatient and emergency room visits since 1995. The DNPR contains records of dates of admission and discharge, discharge diagnoses, and up to 20 secondary discharge diagnosis codes according to the ICD-10 (Schmidt et al. 2015).

The DNHSPD has registered all redeemed prescriptions from pharmacies in Denmark since 2004. The treatments are coded according to the Anatomical Therapeutic Chemical (ATC) classification (Johannesdottir et al. 2012).

### Statistics

An appropriate statistical method to evaluate hospitals’ performance is multilevel models (Abel and Elliott 2019). We used such models to account for the fact that patients were nested within hospitals. Thereby, any unexplained variation in infection was divided into patient-specific variation and hospital-specific variation. Differences among patients were considered by adjusting for the patient-level characteristics: age, sex, comorbidity, BMI, surgery delay, and surgery type.

For each outcome we performed a multilevel Poisson regression analysis, including a random effect to account for the within-cluster correlation between hospitals. Furthermore, an offset term was included for the time parameter; therefore the outcome can be interpreted as a rate (Austin et al. 2018).

League tables for each outcome were created. The league tables show the ranking of hospitals by risk of acquiring a postoperative infection. The crude tables were adjusted for hospital level, while the adjusted tables have taken individual covariates into account. Hospitals with less than 5 outcomes were not shown in the league tables due to Danish data protection agency rules regarding personally identifiable data. However, all hospitals were included in the analyses.

We evaluated hospital variation from the intra-class coefficient (ICC) and the median rate ratio (MRR). ICC denotes the proportion of hospital variance compared with the total variance that is unexplained by the already defined covariates. An ICC value for hospital of 100% denotes all unexplained variation is due to hospital level, while an ICC of 0% denotes all unexplained variation is at patient level. MRR denotes the median relative change in the rate of the outcome between 2 patients with identical characteristics from different hospitals, comparing the highest risk hospital with the lowest risk hospital. An MRR of 1 is equal to no hospital variance. Confidence intervals for ICC and MRR were estimated with bootstrapping using 100 iterations.

For BMI, 17% of data was missing. We applied a multiple imputation strategy, using ordered logistic regression, to impute BMI, assuming data was missing at random, and computed 17 imputations.

### Sensitivity analysis

A series of sensitivity analyses assessed the robustness of our estimates and accounted for variability in clinical practice.

1st, to investigate whether loss to follow-up due to death would introduce bias, we calculated the risk of infection and mortality as a combined outcome. 2nd, patients might already have been infected at admission. Therefore, we repeated the analyses, excluding all patients who had redeemed any antibiotic prescription 14 days prior to surgery date. 3rd, hospitals may have different strategies to identify infections before discharge. Therefore, we investigated whether patients were discharged with infection after the primary hospitalization for hip fracture or readmitted with an infection. 4th, infections may go undetected at the hospital, but later be detected by a general practitioner. Therefore, we combined hospital-treated infection and community-treated infection to a single outcome and repeated the analysis. 5th, to ensure identical follow-up time for community-treated infection, we repeated the analyses starting follow-up at discharge.

All analyses were performed in STATA 15.1 or R version 3.6.1 (StataCorp, College Station, TX, USA).

### Ethics, funding, and potential conflicts of interest

The study was approved by the Danish Data Protection Agency (journal number 2015-57-0002) and Aarhus University’s journal number 2016-051-000001 (record number 880). As there was no contact with patients and no study interventions were performed, permission from the scientific ethical committee was not necessary according to Danish law. The study was supported by a grant from the Novo Nordisk Foundation (reference number NNF190C0056429). The authors reported no conflicts of interest to declare.

## Results

29,598 patients from 23 different hospitals were included, of whom the majority were women and aged 65–89 years. Overall, 15% of patients were diagnosed with a hospital-treated infection, whereas 10% were diagnosed with pneumonia, and 1.8% with sepsis. Additionally, 24% had a community-treated infection within 30 days of surgery (Table 5).

**Table 5.** Patient characteristics. Values are count (%)

	Hospital-treated infection		
	Not infected	Infected	Total
Variable	n = 25,066	n = 4,532	n = 29,598
Women	17,860 (71)	2,694 (59)	20,554 (69)
Age			
65–79	9,670 (38)	1,376 (30)	11,046 (37)
80–89	10,456 (42)	2,092 (46)	12,548 (43)
> 89	4,940 (20)	1,064 (24)	6,004 (20)
Charlson Comorbidity Index			
None (0 points)	9,869 (40)	1,243 (27)	11,112 (37)
Low (1–2 points)	9,844 (39)	1,934 (43)	11,778 (40)
High (> 3 points)	5,353 (21)	1,355 (30)	46,708 (23)
BMI			
Underweight (< 18.5)	3,271 (13)	646 (14)	3,917 (13)
Normal (18.5–24.9)	10,326 (41)	1,726 (38)	12,052 (41)
Overweight (25–29.9)	5,650 (22)	973 (22)	6,623 (22)
Obese (≥ 30)	1,690 (6.7)	314 (6.9)	2,004 (7)
Missing	4,129 (17)	873 (19)	5,002 (17)
Surgery delay			
< 24 h	17,404 (67)	3,002 (66)	20,406 (69)
24–48 h	6,080 (24)	1,230 (27)	7,310 (24)
> 48 h	1,582 (6.3)	300 (6.6)	1,882 (7)
Surgery type			
Osteosynthesis	16,291 (65)	2,782 (61)	19,073 (64)
Total/hemiarthroplasty	8,775 (35)	1,750 (39)	10,525 (36)

### Hospital-treated infections

**Figure 3.**
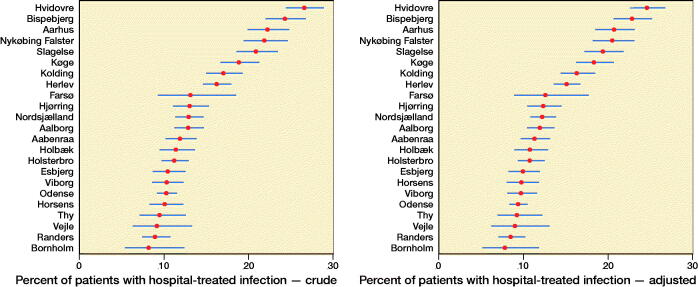
League tables ranking hospitals for hospital-treated infections.

The average risk of any hospital-treated infections varied between 8.2% and 27% among hospitals. After adjustment for hospital case mix, the risk varied from 7.8% to 25% (Figure 3). The adjusted variance attributed to hospital level was 19% (95% CI 10–25). The risk of acquiring any hospital-treated infection at the highest risk hospital compared with the lowest risk hospital for a patient with identical characteristics was 2.0 (CI 1.6–2.3) (Table 6).

**Table 6.** Multilevel Poisson regression for hospital-treated infection and stratified for pneumonia and sepsis. Values are relative risk (95% confidence interval)

Individual variables	Hospital-treated infection	Pneumonia	Sepsis
Sex (ref. Female)			
Male	1.59 (1.50–1.70)	1.81 (1.68–1.95)	2.23 (1.87–2.67)
Age (ref. 65–79)			
80–89	1.40 (1.31–1.50)	1.59 (1.46–1.73)	1.72 (1.40–2.12)
> 89	1.55 (1.43–1.68)	1.84 (1.66–2.03)	1.83 (1.43–2.39)
Charlson Comorbidity Index (ref. 0 points)			
Low (1–2 points)	1.36 (1.27–1.46)	1.48 (1.35–1.61)	1.31 (1.06–1.62)
High (> 3 points)	1.60 (1.48–1.73)	1.68 (1.53–1.86)	1.69 (1.34–2.12)
BMI (ref. 18.5–24.9)			
Underweight (< 18.5)	1.21 (1.11–1.31)	1.28 (1.16–1.41)	1.20 (0.95–1.52)
Overweight (25–29.9)	1.00 (0.93–1.07)	1.00 (0.91–1.09)	0.86 (0.69–1.06)
Obese (≥ 30)	1.11 (0.99–1.24)	1.04 (0.90–1.19)	0.78 (0.55–1.12)
Surgery delay (ref. < 24 h)			
24–48 h	1.09 (1.02–1.17)	1.15 (1.06–1.24)	1.09 (0.89–1.33)
> 48 h	1.06 (0.94–1.19)	1.01 (0.86–1.17)	1.38 (1.00–1.89)
Operation type (ref. Osteosynthesis)			
Total/hemiarthroplasty	1.14 (1.08–1.22)	1.14 (1.06–1.23)	0.93 (0.78–1.12)
Hospital contextual effects			
ICC ^a^ hospital (%)	18.8 (10.0– 24.9)	12.1 (5.7–18.8)	1.8 (0.6–3.7)
MRR ^b^	1.96 (2.33–1.57)	2.08 (0.40–0.97)	1.82 (2.33–1.41)

**^a^**ICC = intra-class coefficient.

**^b^**MRR = median risk ratio.

Furthermore, increasing age and comorbidity were strongly associated with higher risk of hospital-treated infection. Men had an increased risk compared with women (RR = 1.6, CI 1.5–1.7). Underweight patients had a 21% higher risk compared with normal weight patients. Patients operated on with total/hemiarthroplasty had a 14% increased risk compared with patients operated on with osteosynthesis. For the specific hospital-treated infections, pneumonia and sepsis, the results were similar except regarding surgery type (Table 6).

The risk for pneumonia and sepsis varied across hospitals and was reduced after adjustment (Figures 5 and 6, see Supplementary data). The hospital variance for pneumonia and sepsis was similar to any hospital-treated infections. The amount of variation attributed to hospital level was 12% (CI 5.0–15) for pneumonia and 1.8% (CI 0.6–3.7) for sepsis (Table 6).

### Community-treated infections

**Figure 4.**
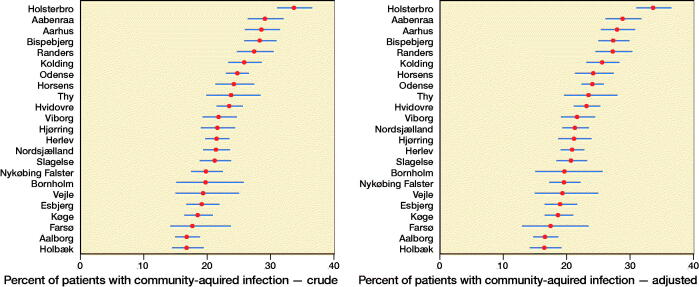
League tables ranking hospitals for community-acquired infections.

The average risk for community-treated infection varied between 17% and 34% among hospitals. After adjustment for hospital case mix, the risk varied from 16% to 34% (Figure 4). The adjusted variance attributed to hospital level was 13% (CI 10–25). The risk of acquiring a community-treated infection at the highest risk hospital compared with the lowest risk hospital for a patient with identical characteristics was 1.4 (95% CI 1.3–1.6) (Table 7).

**Table 7.** Multilevel Poisson regression for community-treated infection. Values are relative risk (95% confidence interval)

Individual variables	Community-treated infection
Sex (ref. Female)	
Male	0.94 (0.89–0.99)
Age (ref. 65–79)	
80–89	1.29 (1.22–1.36)
> 89	1.44 (1.35–1.53)
Charlson Comorbidity Index (ref. 0 points)	
Low (1–2 points)	1.17 (1.10–1.23)
High (> 3 points)	1.24 (1.16–1.32)
BMI (ref. 18.5–24.9)	
Underweight (< 18.5)	1.02 (0.94–1.09)
Overweight (25–29.9)	1.05 (0.99–1.11)
Obese (≥ 30)	1.19 (1.10–1.30)
Surgery delay (ref. < 24 h)	
24–48 h	1.01 (0.96–1.07)
> 48 h	1.04 (0.95– 1.15)
Operation type (ref. Osteosynthesis)	
Total/hemiarthroplasty	0.97 (0.93–1.02)
Hospital contextual effects	
ICC **^a^** hospital (%)	13.3 (6.0–20.5)
MRR **^b^**	1.43 (1.56–1.25)

**^a, b^**See Table 6.

Furthermore, increasing age and comorbidity were strongly associated with higher risk of community-treated infection. Obese patients had a 19% increased risk compared with normal weight patients. There were no differences in the risk of community-treated infection by surgery delay, type of surgery, or gender (Table 7).

### Sensitivity analysis

1st, when combining mortality and hospital-treated infection as a single outcome, the risk varied between 15% and 29% (Table 8, see Supplementary data) with 8.4% (CI 3.8–12) of the variation due to hospital level. The MRR showed an increased risk for a patient operated on at the highest risk hospital of 1.5 (CI 1.3–1.6) compared with the lowest risk hospital. 2nd, excluding all patients who had redeemed a prescription for antibiotics < 14 days prior to surgery did not change the results considerably (Table 9, see Supplementary data). 3rd, three-quarters of hospital-treated infections were detected during primary hospitalization, with hospital variation between 50% and 85%. Hospitals with a high infection risk had more infections detected during primary hospitalization (Figure 7, see Supplementary data). 4th, when combining hospital-treated infection and community-treated infection, the risk of infection was 34%, varying from 25% to 46% between hospitals (Figure 8, see Supplementary data). Hospital level explained 11% (CI 4.1–16) of the variation (Table 10, see Supplementary data). 5th, when starting follow-up at discharge, community-treated infection varied between 15% and 29% (Figure 9, see Supplementary data). The MRR showed an increased risk of 1.3 (CI 1.2–1.5) between the lowest risk hospital and the highest risk hospital. The ICC indicated that 7.3% (CI 3.3–12) of the adjusted variance was due to hospital level (Table 10, see Supplementary data)

## Discussion

Our study is the first to examine the variation between hospitals in the risk of hospital-treated and community-treated infections following hip fracture surgery, and to quantify the hospital-level contribution to the variation using a nationwide population-based cohort design. We found a more than 3-fold difference in hospital-treated infections between hospitals, where 19% of the variation was attributed to hospital level. The variation was sustained when stratifying for pneumonia and sepsis. For community-treated infection, we found a 2-fold difference between hospitals, with 13% of the variation attributed to hospital level.

### Strength and limitations

This study was based on a nationwide population-based cohort design, prospectively collected individual-level data, and complete follow-up of all patients. We included nearly 30,000 patients with free-of-charge and equal access to healthcare services, thereby reducing the risk of selection bias. When investigating death and hospital-treated infection as combined outcome, we found a minor decrease in variation. Therefore, we do not consider loss to follow-up from death to introduce any pertinent bias.

A limitation of this study regards the validity of data, as this is collected by numerous clinicians as part of daily routine clinical work. We cannot exclude the possibility that variation in reporting practice between hospitals can overestimate or underestimate infections. We identified hospital-treated infections based on ICD-10 codes from the DNPR, which is known to have a high positive predictive value (PPV), in other patient groups (Holland-Bill et al. 2014). However, the PPV might vary between hospitals. Unfortunately, we did not have data on radiographs, changes in inflammatory markers etc. to confirm the diagnosis codes’ negative predictive value, and thereby assess the amount of misclassification of infections. However, because infections do not clear spontaneously, we combined hospital-treated infections with community-treated infections and found a slightly lower variation due to hospital level. Additionally, we included only infection diagnoses, for which the hospitals receive payment based on their registration of diagnoses. We therefore assume that all patients treated for infection are registered. Furthermore, in the case of under-reporting infections at specific hospitals, we would have observed some hospitals with a negligible low infection risk, which was not the case. However, when analyzing specific infections such as pneumonia and sepsis we observed a lower variation, as well as a lower amount of variation attributed to hospital level. This points towards the hypothesis that the more severe the infection, the easier the infection becomes to detect, which may lead to less misclassification by hospital variation.

Regarding the infections included in “any infections,” we found that chronic infections such as HIV were very few. The same applies for infections less relevant to hospital admission for hip fracture, such as ear and eye infections (Table 2, see Supplementary data)). Therefore, “any infections” may predominantly be interpreted as infections associated with hip fracture and hospital admission.

Hip fracture patients in Denmark are admitted to the nearest hospital offering hip fracture surgery and are therefore not classified according to health status, fracture severity, or other characteristics. This minimizes the risk of confounding by indication.

Finally, we adjusted for a range of well-established prognostic factors to reduce confounding, including the CCI, which comprised complete in-hospital comorbidity history. However, we did not have information on the severity of diseases in the CCI or full information on all factors exposing for infection. Therefore, we cannot exclude the possibility of residual confounding.

### Comparison with previous literature

Previous studies on hospital variation in postoperative infections have primarily focused on cardiac surgery (Hirahara et al. 2019) or combined multiple surgical procedures (Wakeam et al. 2016). However, 1 study on elective hip and knee arthroplasties has shown a 4-fold difference in risk between hospitals in the United States (Bozic et al. 2014) for complications, including pneumonia. We found a 5-fold difference for pneumonia. However, our study population was acutely operated on, older, more frail, and had more comorbidities compared with patients undergoing elective hip arthroplasty. In addition, our absolute risk estimates were much higher, suggesting that more standardized and complex care of patients could contribute to mortality reduction.

When looking at hospital variation attributed to hospital level in other outcomes, a Dutch study investigated the hospital variation in any-cause readmission within 30 days among patients operated on for a femoral neck fracture. They reported the risk to vary among hospitals between 2.2% and 11% (Hekkert et al. 2018). Moreover, the study found 2.3% of the variation explained by hospital level. We found a higher risk only of postoperative infections, probably due to our inclusion of infections detected during primary hospitalization. Furthermore, hospital level explained 19% of the variation in postoperative infections in our study. This suggest that variation due to hospital level for postoperative infections is more frequent than for any-cause readmission. Any-cause readmissions are thereby a less sensitive marker for hospital performance than postoperative infections. The same applies to mortality. This is supported by the fact that the ICC and MRR in our study is higher compared with a previous Danish variation study on 30-day mortality after hip fracture, which found that less than 1% of the variation in mortality was explained by hospital level (Kristensen et al. 2019).

### Clinical implications

Our results imply that quality of in-hospital care for hip fracture patients is not homogeneous regarding postoperative infections. We found that patients predominantly had their infection detected during the primary hospitalization. We found nearly a 5th of the variation was explained by hospital-level factors, whereas the largest variation was due to individual-level factors. Previous studies have evaluated interventions to decrease postoperative pneumonia with success. As we showed the most common postoperative infection to be pneumonia, this should be the primary focus in such interventions. Kazaure et al. (2014) propose a standardized postoperative pneumonia program, including education of nursing staff, coughing and deep-breathing exercises, twice-daily oral hygiene, ambulation, and elevated head of the bed during meals. This intervention showed a 44% decreased rate of postoperative pneumonia among 4,099 American, non-cardiac, surgical patients. Furthermore, a study from Taiwan included 240 hip fracture patients. They showed a pneumonia risk of 14%, which we regard as comparable to ours at 10% (Chang et al. 2018). Their study showed a decrease in postoperative pneumonia to 5.9% among an intervention group implemented with deep-breathing exercises, chest physiotherapy, and cough-assisted maneuvers. Since postoperative infections are associated with higher mortality, a decrease in postoperative infection would lead to decreased mortality. In conclusion, we advocate for improvement of national clinical guidelines to detect and treat infections during primary hospitalization. This may be as a standardized infection screening of all hip fracture patients or implantation of a standardized infection prevention program.

## Supplementary data

Tables 1–4 and 8–10 and Figures 1–2 and 5–10 are available as supplementary data in the online version of this article, http://dx.doi.org/10.1080/17453674.2020.1863688
